# Perspectives on Continuous Glucose Monitoring Among Adults with Type 2 Diabetes in the United Kingdom: Cross-Sectional Survey

**DOI:** 10.2196/89898

**Published:** 2026-06-26

**Authors:** Albandari Alharbi, Elena Lammila-Escalera, Benedict Hayhoe, Reham Aldakhil, Azeem Majeed, Austen El-Osta, Ana Luisa Neves

**Affiliations:** 1 Department of Primary Care and Public Health School of Public Health Imperial College London London, London United Kingdom

**Keywords:** continuous glucose monitoring, CGM, type 2 diabetes, T2D, diabetes technology, digital health, diabetes self-management

## Abstract

**Background:**

Type 2 diabetes (T2D) is one of the most common noncommunicable diseases, requiring ongoing lifestyle changes and continuous glucose management through medication, diet, and physical activity. Traditional self-monitoring of blood glucose can be burdensome, especially with frequent finger pricks. As continuous glucose monitoring (CGM) becomes more affordable and accessible, it offers benefits such as increased glucose awareness, behavioral modifications, and reduced anxiety. However, challenges remain, including cost, discomfort, skin reactions, and privacy concerns. In the United Kingdom, perceptions of CGM among people with T2D, including both users and nonusers, are not well understood, limiting insight into factors influencing adoption and sustained use.

**Objective:**

This study aims to explore how adults with T2D perceive the benefits and challenges of using CGM, including both current users and nonusers.

**Methods:**

This study used a cross-sectional, online survey using YouGov’s nationally representative panel to explore experiences of CGM among adults with T2D in the United Kingdom. A total of 531 participants were recruited from November to December 2024. Thematic analysis of responses to 2 open-ended questions identified key perceived benefits and challenges associated with CGM use.

**Results:**

A total of 531 adults with T2D completed the YouGov online survey. Over half were male (297/531, 55.9%) and aged 65 years and older (281/531, 52.9%). Two-thirds (347/531, 65.3%) had lived with T2D for more than 5 years, and 9.6% (51/531) use or had previously used a CGM. Overall, 50.8% (270/531) responded to at least one free-text question, with 49% (260/531) commenting on benefits and 33.1% (176/531) on challenges. Thematic analysis identified five key benefit themes: (1) reduced monitoring burden, described as eliminating frequent finger prick testing and simplifying daily routines; (2) lifestyle feedback, enabling participants to better understand how diet and physical activity influence glucose levels; (3) greater control, by supporting more informed decision-making and increasing confidence in self-management; (4) feeling safer, through alerts for hypo- and hyperglycemia; and (5) sharing data with clinicians, which facilitated communication and more collaborative care. The main challenges were (1) access barriers, including restrictive eligibility criteria and the high cost of self-funding; (2) device issues, such as discomfort, inconvenience, and practical difficulties wearing the sensor; (3) technology reliance, with concerns about depending on devices rather than listening to bodily cues; (4) emotional strain, including anxiety, over-monitoring, and increased preoccupation with glucose levels; and (5) data concerns, particularly regarding accuracy, interpretation, and privacy.

**Conclusions:**

Adults with T2D, including both users and nonusers, described CGM as a practical and empowering tool that improves understanding, safety, and collaboration with health care providers. Nevertheless, access barriers, usability issues, and emotional and data-related burdens remain major obstacles to equitable adoption. Addressing these through improved affordability, digital literacy support, and customized clinical guidance may support ongoing and inclusive CGM use in routine care.

## Introduction

Type 2 diabetes (T2D) is a growing global health challenge, affecting approximately 10.5% of the world's population [1,2]. In the United Kingdom, around 12.1 million adults are living with diabetes or prediabetes, placing a substantial economic burden on the National Health Service (NHS), accounting for around 6% of the total NHS budget for managing preventable complications [3-5]. Effective diabetes management relies on sustained self-management, primarily the regular monitoring of blood glucose levels [6].

Self-monitoring of blood glucose (SMBG) is widely used but often poorly adhered to, even when devices are available [7-13]. Qualitative research suggests that individuals face multiple barriers to regular SMBG, including pain, inconvenience, and emotional burden, such as frustration, anxiety, and self-blame [8,9,14]. These challenges can reduce motivation and contribute to disengagement from routine monitoring, particularly when individuals feel unsupported [15].

Continuous glucose monitoring (CGM) has emerged as an alternative approach that may address some of these limitations by providing real-time glucose data and reducing the need for frequent finger prick testing. This sensor-based system measures interstitial glucose levels via a subcutaneously inserted filament and transmits the data to a receiver or a smartphone, providing the user with trend arrows and alerts for hypo- or hyperglycemia to facilitate proactive self-management [10-12].

Over the past decade, CGM devices have become smaller, more affordable, and increasingly user-friendly [13]. Preliminary research indicates that CGM offers significant benefits over SMBG for individuals with T2D, including greater reductions in hemoglobin A_1c_ levels and fewer hospitalizations due to hypoglycemia [15,16]. Beyond glycemic outcomes, CGM may also reduce the burden of diabetes self-management by providing a more responsive, person-centered approach to glucose monitoring [17,18]. Qualitative evidence suggests that CGM can enhance self-management, improve understanding of the impact of lifestyle behaviors, and support engagement with care, although concerns remain regarding cost, usability, and device burden [19,20]. These findings indicate that experiences of CGM are shaped not only by the technology itself but also by individual and contextual factors.

In the United Kingdom, access to CGM through the NHS remains restricted for adults living with T2D and is typically limited to those with specific clinical indications, such as intensive insulin use or recurrent hypoglycemia [21,22]. As a result, many individuals who may benefit from CGM are unable to access it without self-funding, contributing to variation in uptake and equity of access across the population. Earlier and more equitable access to CGM may support self-management and treatment optimization, but understanding how people perceive its value and burden is crucial for integrating it into routine care [23].

Despite growing interest in CGM, most existing research has focused on current CGM users, often within small or clinically selected samples. The perspectives of nonusers, including those who have never used CGM but might benefit from it, remain underexplored. Understanding these perspectives is critical for identifying barriers to uptake and informing equitable implementation in routine care. This study aimed to explore the perceived benefits and challenges of CGM among adults with T2D, including both users and nonusers, using a cross-sectional survey with qualitative analysis of open-ended responses.

## Methods

### Overview

This online cross-sectional survey was conducted among the public. While online surveys were historically underused due to the dominance of interviews and focus groups in qualitative research and misplaced assumptions about insufficient data depth, they are now a recognized method in qualitative research [24]. Qualitative surveys typically use open-ended questions to produce long-form answers that capture personal narratives and experiences. The CHERRIES (Checklist for Reporting Results of Internet E-Surveys) and the COREQ (Consolidated Criteria for Reporting Qualitative Research) checklists were used to ensure that the study met the recommended standards of survey and qualitative data reporting (Multimedia Appendix 1) [25,26].

### Study Population and Sampling

Participants were recruited via YouGov, an international online research data and analytics technology group [27]. A nationally representative sample was recruited using a quota sampling approach to ensure representativeness by age, gender, ethnicity, social grade, and region within the UK. This was complemented by targeted sampling of individuals known to have T2D, drawn to be representative of those with T2D by age, gender, and ethnicity within the United Kingdom [28]. Although the survey included both general-population respondents and individuals with self-reported T2D, the present analysis focuses only on the T2D subsample.

Eligibility criteria included being an adult (aged ≥18 years) with a T2D diagnosis (self-identified). We did not require prior familiarity with CGM as an eligibility criterion. Instead, participants were asked directly if they had ever used CGM, enabling us to classify respondents as users or nonusers. Additionally, individuals had to be UK residents and able to complete an online survey in English. Potential respondents were contacted by email and invited to participate in the online survey via the YouGov platform, which supports completion using smartphones, tablets, or desktops. Respondents were not informed of the survey topic in the email to avoid participation bias. The survey was live from November 22, 2024, to December 4, 2024, and responses were anonymized before the research team received them. YouGov conducted automated completeness and quality checks before delivering the data, excluding submissions that did not pass them.

The final dataset comprised 1525 completed responses (overall completion rate 89.2%). In the T2D subsample, 553 panel members accepted the invitation; 18 (3.3%) did not complete the questionnaire, and 4 (0.7%) were excluded during quality checks, yielding a final analytic sample of 531 participants (completion among accepters: 96%). Owing to the online format, reasons for drop-out were not ascertainable. Respondents received standard YouGov panel incentives.

### Description of Questionnaire

The questionnaire consisted of 66 items assessing the experiences of individuals with T2D in the context of adopting digital technologies, including CGM, for self-management (Multimedia Appendix 2). The questionnaire included 5-point Likert-scale items (ranging from “strongly disagree” to “strongly agree”) for the eHealth Literacy Scale (eHEALS) and theoretical constructs, multiple-choice questions for demographic and clinical characteristics, and open-ended items for qualitative responses. The estimated completion time was 10-15 minutes. Items were presented in a fixed order for all respondents, without randomizing the order, and the format did not allow respondents to revisit or edit their previous responses. Participants’ characteristics were collected, including gender, country, setting, educational level, ethnicity, age, time since diagnosis of T2D, diabetes history, and CGM use. All respondents received the complete set of questionnaire items, regardless of whether they used CGM. Perceptions regarding the main benefits and challenges of using CGM were specifically assessed using two free-text questions: (1) "Based on what you've seen or heard about Continuous Glucose Monitoring or your personal experience, what are the main benefits of Continuous Glucose Monitoring?," and (2) "What are the main challenges of Continuous Glucose Monitoring?" This study analyzed these two open-ended questions on the perceived benefits and challenges of technology use to elicit participants’ subjective experiences and contextual factors influencing adoption and engagement. These items were informed by established technology acceptance and behavior-change frameworks, including the Technology Acceptance Model [29], the Unified Theory of Acceptance and Use of Technology [30], and Social Cognitive Theory [31], which collectively emphasize that both facilitators and barriers jointly shape technology uptake and sustained use.

The survey was created by a multidisciplinary team including medical doctors (ALN, AM, and BH), a nurse (AA), and a health services researcher (AE-O), all experienced in questionnaire development and administration. The survey items were developed based on existing literature, and each question was mapped to one or more theoretical frameworks, including Knowledge, Attitudes, and Practices; Capability, Opportunity, Motivation-Behavior; and Unified Theory of Acceptance and Use of Technology [30,32,33]. To ensure the instrument’s clarity and relevance, the survey was reviewed by the survey development team and YouGov and piloted internally.

### Data Analysis

Descriptive statistics were used to summarize demographic characteristics, including gender, country, setting, educational level, ethnicity, age, diabetes history, and history of CGM use. No poststratification weighting was applied as representativeness was addressed through the quota sampling design at recruitment. Two optional open-ended questions (perceived benefits of CGM and perceived challenges) were analyzed using thematic analysis [34]. Of 531 participants, 260 (49%) provided a free-text response on the perceived benefits of CGM, 176 (33.1%) on the perceived challenges of CGM, 166 (31.3%) answered both, 104 (19.6%) answered one of them, and 261 (49.2%) answered neither. The thematic analysis included all available free-text responses from both CGM users and nonusers and was not restricted to CGM users.

Thematic analysis of the free-text responses was performed in Microsoft Excel using Braun and Clarke’s approach. This approach follows six key stages: familiarization with the data, generation of initial codes, searching for themes, reviewing themes, defining and naming themes, and producing the report [35]. The coding framework incorporated both deductive and inductive elements, allowing emergent themes to be continuously integrated into the analysis. Deductive categories were informed by previous literature on CGM use and digital self-management, while inductive coding captured novel or emergent ideas. Two researchers (AA and RA) independently coded each response; disagreements were resolved through discussion, and, if necessary, a third researcher (ALN) adjudicated the final code. Data saturation was achieved, and no new themes were identified toward the conclusion of the analyses. As participants did not consent to further contact, it was not possible to request feedback on the findings.

We derived CGM status from prior CGM use (Yes or No). Responses marked “yes” were treated as lived experience (user), whereas responses marked “no” were treated as hypothetical or anticipated experience (nonuser). Each coded excerpt was tagged with CGM status, and this tagging was used for within- and between-group comparisons; quotations in the Results are labeled accordingly.

### Ethical Considerations

This study was a secondary analysis of free-text responses from a previously conducted survey. Ethical approval was obtained from Imperial College Ethics Research Committee (Reference 7373621). All participants in the original survey provided informed consent, including permission for their responses to be used in future secondary analyses, in accordance with YouGov procedures. No additional consent was required for the current study. The dataset analyzed was fully anonymized prior to secondary analysis, with no identifying information retained. Free-text responses were reviewed to ensure the removal of any inadvertent identifiers. All reporting was based on anonymized, de-identified text responses. Participants received compensation for their time in line with usual YouGov procedures.

## Results

### Participants’ Characteristics

A total of 531 adults with T2D completed the online survey and were included in this analysis. Of these, 55.9% (297/531) were male, and 52.9% (281/531) were aged 65 years and older. Most lived in England (444/531, 83.7%), followed by Scotland (45/531, 8.5%), Wales (28/531, 5.2%), and Northern Ireland (14/531, 2.6%). Most participants (405/531, 76.3%) lived in urban settings. In terms of ethnicity, 76.6% (407/531) identified as Non-Hispanic White, while 23.4% (124/531) identified as belonging to an ethnic minority group. Regarding educational attainment, 57.1% (303/531) had less than degree-level education, 18.2% (97/531) held an undergraduate degree, and 22.8% (121/531) had a postgraduate degree or higher.

More than half of the participants, 65.3% (347/531), reported having T2D for more than 5 years, while 14.9% (79/531) had been diagnosed within the past 3 to 5 years. Only 9.2% (49/531) reported having been diagnosed within the last year. Regarding CGM use, 9.6% (51/531) reported using CGM. Among CGM users, 37.3% (19/51) had been using it for more than 6 months, and 27.5% (14/51) had been using it for less than a month. A full description of the participants is shown in Table 1.

**Table 1:** Characteristics of the study sample of adults with type 2 diabetes in the United Kingdom (N=531).

Characteristics			Values, n (%)
**Sex**			
	Female	234 (44.1)	
	Male	297 (55.9)	
	Total	531 (100)	
	Missing	0 (0)	
**Age (years)**			
	65+	281 (52.9)	
	45-64	207 (38.9)	
	18-44	44 (8.3)	
	Missing	0 (0)	
**Country**			
	Wales	28 (5.2)	
	Scotland	45 (8.5)	
	Northern Ireland	14 (2.6)	
	England	444 (83.7)	
	Missing	0 (0)	
**Setting**			
	Urban	405 (76.3)	
	Town and fringe	55 (10.4)	
	Rural	47 (8.8)	
	Missing	14 (2.7)	
**Ethnicity**			
	Non-Hispanic White	407 (76.6)	
	Ethnic minority	124 (23.4)	
	Missing	0 (0)	
**Educational level**			
	Below degree level	303 (57.1)	
	Undergraduate degree	97 (18.2)	
	Postgraduate degree or above	121 (22.8)	
	Missing	10 (2)	
**Duration since T2D^a^** **diagnosis**			
	Less than 1 year	49 (9.2)	
	1-2 years	53 (9.9)	
	3-5 years	79 (14.9)	
	More than 5 years	347 (65.3)	
	Prefer not to say	4 (0.8)	
	Missing	0 (0)	
**History of CGM^b^** **use**			
	Yes	51 (9.6)	
	No	351 (66.1)	
	Missing	129 (24.3)	
**Duration of CGM use**			
	Less than a month	14 (27.5)	
	1-3 months	7 (13.7)	
	4-6 months	6 (11.8)	
	More than 6 months	19 (37.3)	
	Missing	5 (9.8)	

^a^T2D: type 2 diabetes.

^b^CGM: continuous glucose monitoring.

### Main Benefits

#### Overview

Participants reported various benefits of CGM use, which were clustered into five overarching themes: (1) reduced monitoring burden, (2) lifestyle feedback, (3) greater control, (4) feeling safer, and (5) sharing data with clinicians. A comprehensive overview of the themes and associated subthemes is provided in Table 2.

**Table 2:** Perceived benefits of continuous glucose monitoring among the study sample of adults with type 2 diabetes in the United Kingdom (N=531).

Themes and subthemes		Quotation
**Reduced monitoring burden**		
	Ease of use	“Ease of use and continuous monitoring.” (ID619, Nonuser) “Easy control.” (ID118, Nonuser) “Ease of mind.” (ID1531, Nonuser)
	Convenience	“No need to carry around a testing kit. connects to your phone for instant readouts.” (ID1646, Nonuser) “Saves the hassle of having your testing kit with you at all times and inconvenience.” (ID1696, Nonuser)
	Reduced discomfort	“No finger pricking for blood testing...” (ID1472, Nonuser) “No need to take blood samples.” (ID923, Nonuser)
**Lifestyle feedback**		
	Access to continuous, real-time data	“You will know, in real time, your blood sugar level.” (ID1682, Nonuser) “Instant information to manage diabetes.” (ID784, User)
	Feedback on how lifestyle impacts glucose levels	“Direct feedback on effect of eating certain foods on blood sugar levels.” (ID547, User) “Understand your glucose levels effectively.” (ID184, User) “Knowing your glucose levels before during and after meals.” (ID825, Nonuser)
**Greater control**		
	Medication optimization	“You will know, in real time, your blood sugar level. This will allow you to take corrective measures immediately, should they be required.” (ID1862, Nonuser) “Control of insulin dosage according to glucose levels.” (ID616, Nonuser)
	Lifestyle behavior optimization	“Enables you to make real time decisions about diet and exercise to keep my sugar levels in the healthy range.” (ID1461, User)
	Increased patient empowerment	“Help managing your diabetes independently.” (ID898, Nonuser) “Responsive 24 hours a day allowing individual to take ownership of their illness.” (ID1648, Nonuser)
**Feeling safer**		
	Improved safety through alerts	“Immediately alerted.” (ID1539, Nonuser) “There is an alarm should levels drop or peak which potentially can save lives.” (ID1554, Nonuser) “Warnings on your phone when levels are out.” (ID1681, Nonuser)
	Prevention of hypo or hyperglycemia episodes	“It prevents disorders and accidents that can result from abnormal glucose levels.” (ID554, User) “Avoiding hypos.” (ID1423, Nonuser)
Sharing data with clinicians		“(...) results available to share with healthcare providers.” (ID1541, Nonuser) “(...) monitor results would be available to a health care professional to advise on treatment.” (ID1544, Nonuser)

#### Reduced Monitoring Burden

Reducing monitoring burden emerged as a key advantage of CGM, with participants highlighting its (1) ease of use, (2) convenience, and (3) reduced discomfort. Participants reported that the devices were intuitive and easy to use, and participants appreciated the convenience of not having to carry a traditional glucose testing kit. Some participants also valued the reduced discomfort from eliminating the need for frequent finger prick testing.

#### Lifestyle Feedback

Participants found that the ability to access continuous real-time data was particularly valuable, as it allowed them to better understand how their lifestyle choices affected their glucose levels and recognize patterns over time. Some noted that real-time feedback enabled them to identify triggers and make timely adjustments to their habits, improving glucose management and helping prevent glucose fluctuations.

#### Greater Control

Participants reported that CGM helped, or could help, them take ownership of their condition through (1) medication optimization, (2) lifestyle behavior optimization, and (3) overall increased patient empowerment. Some respondents noted that CGM enabled more precise insulin dosing and overall medication optimization. The ability to track glucose fluctuations was also deemed to support lifestyle behavior change, helping individuals make informed choices about diet and exercise and helping them to achieve more stable glucose levels. Participants also emphasized that CGM enhanced their sense of control and confidence, promoted greater independence in managing their condition, and allowed them to take a more active role in their diabetes care.

#### Feeling Safer

The participants reinforced the key role of the integrated alert system, which provided an immediate warning when glucose levels became too high or too low. Participants described how these real-time alerts improved their ability to act swiftly, potentially preventing dangerous situations and reducing the risk of hypo- and hyperglycemic episodes.

#### Sharing Data With Clinicians

Another major benefit identified was the ability to share data with health care providers. The ability to share real-time glucose data facilitated more effective communication and informed treatment adjustments. Participants also highlighted that this improved exchange of information helped them feel more supported in managing their condition and strengthened collaboration between patients and health care professionals.

### Main Challenges

#### Overview

Participants noted five main challenges associated with the use of CGM: (1) access barriers, (2) device issues, (3) technology reliance, (4) emotional strain, and (5) data concerns. A detailed overview of the themes and their corresponding subthemes is given in Table 3.

**Table 3:** Perceived challenges of continuous glucose monitoring among the study sample of adults with type 2 diabetes in the United Kingdom (N=531).

Challenges		Quotations
**Access barriers**		
	Prescription barriers	“Doctor's not letting me have one due to being type 2, even though I'm on medication that causes me to have hypos.” (ID1214, User) “Getting it prescribed. I'm Diabetic and not heavy enough for NHS^a^ help, it’s like they want type 2 diabetics to get enormously overweight or die.” (ID1333, User)
	The high cost of self-funding	“£120 month is out of my price range.” (ID1333, User) “Would like to use them all the time but find them too expensive at approximately £50 for 2 weeks monitoring.” (ID574, User) “Getting one. Cost.” (ID822, Nonuser)
**Device issues**		
	General usability issues	“It is on the upper arm... it could be knocked or caught on something, and you could have difficulty in moving your arm.” (ID861, User) “Having something constantly attached to the arm.” (ID953, Nonuser) “Applying the device.” (ID1555, User)
	Pain and discomfort	“Pain and uncomfortable of the pin that goes in your arm. Catching the monitor when showering/ changing etc.” (ID1279, Nonuser) “It is still a pointy needle sticking in your arm!” (ID1496, Nonuser) “Pain.” (ID1536, Nonuser)
	Technological issues	“Constant use of cell phone.” (ID1985, Nonuser) “Doesn't always stay connected to my phone. Often has sensor issues. Getting faulty sensors. iPhone doesn't alarm if wearing Apple Watch.” (ID1759, User)
	Technology reliance	“Become more reliant on tech, rather than pay attention to your body response.” (ID1638, Nonuser) “Being totally obsessed with the state of your health and an inability to trust your own judgement as to the state of your health.” (ID1763, Nonuser)
	Emotional strain	“(...) potential to be obsessive about checking and diet etc.” (ID615, Nonuser) “Getting too preoccupied with them.” (ID1586, Nonuser)
**Data concerns**		
	Data irrelevance and inaccuracy	“Irrelevant results.” (ID843, Nonuser) “Accuracy (issues).” (ID643, User)
	Privacy concerns	“Too much information, unsure on how secure they are.” (ID1050, Nonuser) “Invasion of privacy.” (ID1690, Nonuser) “(...) big brother monitoring by doctors and possibly others.” (ID1043, Nonuser)

^a^NHS: National Health Service.

#### Access Barriers

Access to CGM was reported as a significant challenge, either due to (1) prescription barriers to these devices or (2) the high cost of self-funding. Participants expressed frustration with restrictive eligibility criteria for CGM coverage, feeling unfairly excluded and forced to rely on self-funded purchases. For those who did not qualify for coverage, the financial burden of CGM was a major concern. Many described it as too expensive for regular use, with some stating that they could only afford it occasionally.

#### Device Issues

Participants’ responses identified several usability and technological challenges associated with CGM use, broadly grouped into three main areas: (1) general usability issues, (2) pain and discomfort, and (3) technological issues. Regarding general usability, participants raised concerns about the physical placement of CGM devices and how this placement affected daily activities. Some participants found CGMs inconvenient during activities such as showering, exercising, or wearing certain clothing; others expressed frustration with the device detaching unexpectedly. Some participants described experiencing discomfort, including skin irritation or difficulties in keeping the device securely attached. Participants also expressed a range of concerns about the technological challenges associated with CGM, primarily regarding calibration requirements, sensor accuracy, and connectivity issues.

#### Technology Reliance

Participants expressed concerns that CGM might lead to an overreliance on technology, diverting their attention from their own bodily responses and judgment.

#### Emotional Strain

The emotional burden of using CGM was highlighted, as some participants reported increased anxiety and being overly focused on their glucose levels, as well as their lifestyle behaviors.

#### Data Concerns

Data-related issues were noted at three levels: (1) data irrelevance and inaccuracy, (2) privacy concerns, and (3) difficulties discussing data with health care professionals. Concerns about CGM accuracy and relevance were noted, with participants citing issues such as calibration requirements and lag time, which sometimes resulted in unreliable readings. Some believed that occasional inaccuracies in CGM data led to unnecessary concerns or incorrect treatment decisions. Privacy concerns were raised regarding data security, with participants feeling uneasy about how their glucose information was stored and shared, or worried about being monitored without their consent.

## Discussion

### Principal Findings

Participants with T2D found CGM to be a practical and supportive tool that improved their understanding of glucose patterns and helped with self-management. Many noted the importance of real-time feedback for linking lifestyle choices to glucose changes, as well as the comfort provided by continuous monitoring and alert systems. Nevertheless, they also reported barriers such as limited access within the NHS, financial costs of self-funding, usability and technological issues, emotional stress from constant data exposure, and worries about data privacy. Similar perceptions were expressed by both CGM users and nonusers, suggesting that expectations and lived experiences shape how the technology is understood. Overall, the findings indicate that while participants often view CGM positively, its uptake may be influenced by a combination of structural constraints and individual-level factors.

### Comparison With Previous Literature

#### Benefits

Reduced monitoring burden emerged as a key perceived benefit. Participants highlighted the convenience of continuous monitoring and the elimination of frequent finger prick testing, consistent with previous evidence that CGM reduces the physical and psychological burden of SMBG and improves treatment satisfaction [34,36,37]. This perceived relief was also evident among nonusers, suggesting that anticipated reductions in burden may shape interest in CGM prior to adoption.

From a behavioral perspective, these findings align with the Capability, Opportunity, Motivation-Behavior model, whereby reducing discomfort and inconvenience increases physical opportunity and supports automatic motivation [19,33]. In this context, CGM may not only facilitate behavior but also act as a “cue to action,” particularly for individuals who perceive traditional monitoring as burdensome [19].

Participants also emphasized the value of real-time feedback in linking lifestyle behaviors to glucose fluctuations. Consistent with prior research, CGM may enhance psychological capability and reflective motivation, shifting diabetes management from reliance on general advice to a more individualized, data-driven process [38]. Perceived improvements in safety were another prominent benefit. Participants described alerts for hypo- and hyperglycemia as reassuring, enabling earlier detection of adverse events. This aligns with clinical evidence showing that alert-enabled CGM systems can reduce hypoglycemia exposure and improve time in range [39,40]. Importantly, these perceived benefits were reported by both users and nonusers, suggesting that expectations of reassurance may shape attitudes toward adoption.

Participants also reported that CGM data could support more productive interactions with health care professionals. The ability to review glucose trends collaboratively may facilitate shared decision-making and reduce reliance on retrospective indicators such as hemoglobin A_1c_. Previous research similarly shows that standardized CGM outputs, such as the ambulatory glucose profile, support pattern recognition and more personalized treatment adjustments during clinical consultations [41-43]. Together, these findings position CGM not only as a self-management tool but also as a resource that can enhance patient-clinician communication.

#### Challenges

Despite these perceived benefits, participants also identified several barriers that may limit adoption. Restricted eligibility criteria within the NHS were frequently described as a key obstacle, reflecting broader evidence of variation and inequities in CGM prescribing practices [44-46]. These results illustrate how structural constraints to CGM access are experienced at the patient level, particularly among individuals who perceive themselves as suitable candidates for the technology [33].

Cost was another reported concern. For those not eligible for publicly funded devices, the ongoing cost of CGM devices was perceived as prohibitive, consistent with previous research highlighting affordability as a major barrier to uptake, particularly among lower socioeconomic groups [19,33]. This suggests that financial constraints may deter adoption, even where perceived benefits are high. Usability challenges were also reported, including discomfort, device placement issues, and connectivity problems. Similar barriers have been documented in qualitative studies, in which skin irritation, sensor adhesion problems, and device inconvenience were identified as barriers to sustained use [47,48]. In addition, technological complexity may place demands on psychological capability, particularly for individuals with lower digital literacy, highlighting the importance of support for effective use [49].

Participants also expressed concern about becoming overly reliant on CGM, describing a shift away from bodily awareness toward technology-dependent monitoring. This tension between automated data and personal judgment has been observed in another study examining diabetes technologies, suggesting that increased monitoring may not always translate into greater confidence or autonomy [50]. The emotional impact of CGM was also mixed. While some participants reported reassurance and increased confidence, others reported anxiety, preoccupation, or potential over-monitoring. This reflects a growing body of literature indicating that CGM can both alleviate and exacerbate emotional burden, depending on individual context and experience [51,52]. Issues such as data overload and alert fatigue may further contribute to these experiences.

Finally, concerns regarding data reliability and privacy were identified. Uncertainty about the reliability of readings and the security of personal health data may influence trust in CGM systems [19,53]. Similar concerns have been reported in broader digital health research, particularly regarding data ownership and potential third-party access [54]. In this context, trust in both the device and the wider data infrastructure may play an important role in shaping engagement with CGM.

### Study Limitations

This study has several limitations. For instance, as an online survey, it may have underrepresented individuals with limited digital access or lower digital literacy, which is particularly relevant given the study’s focus on a digital health technology. In addition, the qualitative component relied on responses to two open-ended questions, which limited the depth of insight compared with interviews or focus groups. The absence of triangulation with additional qualitative methods or data sources may further reduce the depth and credibility of the identified themes. Although the survey instrument was tested internally to assess clarity and flow, a formal assessment of inter-rater reliability was not undertaken, which may limit the evaluation of consistency in how the questions were interpreted.

The composition of the sample should also be considered when interpreting the findings. The predominance of nonusers means that many responses reflect anticipated perceptions rather than the lived experience of CGM use. Furthermore, diabetes status was self-reported because the study was conducted outside a clinical setting and without access to medical records. While this approach is common in population-based research, some degree of misclassification is possible. Self-reported responses may also be subject to social desirability bias, and nonresponse bias may have occurred if individuals experiencing greater distress or barriers to care were less likely to participate. Finally, the survey did not capture information on the specific CGM devices used by participants, limiting the ability to explore how device characteristics, such as cost, wear time, or usability, may have influenced perceptions.

### Conclusions

This study contributes to the growing literature on CGM in T2D by providing qualitative insight from a nationally representative UK sample, including both users and nonusers. By capturing perspectives across the adoption pathway, the findings highlight not only the lived experiences of CGM use but also the anticipated benefits and pre-adoption concerns that are often overlooked in user-focused research. In doing so, the study extends current understanding by showing that perceptions of CGM are shaped as much by expectations and contextual constraints as by direct experience.

A key implication is that successful implementation of CGM depends not only on the clinical effectiveness of the technology but also on how it is experienced in everyday life. Barriers related to access, affordability, usability, and data interpretation remain central to how CGM is perceived and adopted, underscoring the importance of addressing both structural and user-level factors in parallel. Future research should build on these findings by incorporating mixed-method approaches to develop an understanding of patient experiences and by examining how perceptions vary across population subgroups and stages of adoption. These designs may also help assess the prominence of identified themes and explore differences between users and nonusers. In addition, future studies should consider the influence of device-specific characteristics and, where feasible, incorporate clinically verified measures to strengthen validity.

These findings emphasize that expanding access to CGM must be accompanied by strategies that support equitable uptake and sustained use. This includes clearer, more inclusive prescribing pathways, efforts to reduce financial barriers, and tailored education and support to help individuals interpret and act on CGM data. Without such measures, there is a risk that wider implementation of CGM may reinforce, rather than reduce, existing inequalities in diabetes care.

## Appendix

Multimedia Appendix 1 CHERRIES/COREQ checklists.

Multimedia Appendix 2 Survey questions.

## Abbreviations

CGM: Continuous Glucose Monitor
CHERRIES: Checklist for Reporting Results of Internet E-Surveys
COREQ: Consolidated Criteria for Reporting Qualitative Research
NHS: National Health Service
SMBG: Self-Monitoring of Blood Glucose
T2D: Type 2 Diabetes

## Data Availability

The data generated and analyzed during this study are not publicly available because public data sharing was not included in the participant consent process and is not permitted under the study’s ethical approval arrangements. Further information about the study materials may be available from the corresponding author upon reasonable request, subject to ethical and institutional approval.
