# Supplementary material for: Perspectives on Continuous Glucose Monitoring Among Adults with Type 2 Diabetes in the United Kingdom: Cross-Sectional Survey
Source: JMIR Form Res. 2026 Jun 26;10:e89898. doi: 10.2196/89898 (PMC13354950; doi:10.2196/89898)
Supplement: Multimedia Appendix 1 [file formative_v10i1e89898_app1.pdf]

## The Checklist for Reporting Results of Internet E-Surveys (CHERRIES)

| Item Category | Checklist Item | Explanation | Location/Notes |
|---|---|---|---|
| **Design** | Describe survey design | Describe target population, sample frame. Is the sample a convenience sample? (In “open” surveys this is most likely.) | Pg. 6-7. Nationally representative YouGov panel using quota sampling by age, gender, ethnicity, social grade and region. T2D subsample also drawn to be representative. |
| **IRB (Institutional Review Board) approval and informed consent process** | IRB approval | Mention whether the study has been approved by an IRB. | Pg. 10. Imperial College Ethics Research Committee approval, Ref. 7373621. |
| | Informed consent | Describe the informed consent process. Where were the participants told the length of time of the survey, which data were stored and where and for how long, who the investigator was, and the purpose of the study? | Pg. 10. All participants provided informed consent including permission for secondary analysis, per YouGov procedures. No additional consent required for secondary analysis. |
| | Data protection | If any personal information was collected or stored, describe what mechanisms were used to protect unauthorized access. | Pg. 10. Dataset fully anonymised prior to secondary analysis. Free-text responses reviewed to remove inadvertent identifies. No identifying information retained. |
| **Development and pre-testing** | Development and testing | State how the survey was developed, including whether the usability and technical functionality of the electronic questionnaire had been tested before fielding the questionnaire. | Pg. 8. Multidisciplinary team developed items mapped to theoretical frameworks (KAP, COM-B, UTAUT2) Reviewed by YouGov and piloted internally. |
| **Recruitment process and description of the sample having access to the questionnaire** | Open survey versus closed survey | An “open survey” is a survey open for each visitor of a site, while a closed survey is only open to a sample which the investigator knows (password-protected survey). | Pg. 6-7. Closed via YouGov panel, respondents were known YouGov panel members. |
| | Contact mode | Indicate whether or not the initial contact with the potential participants was made on the Internet. (Investigators may also send out questionnaires by mail and allow for Web-based data entry.) | Pg. 6-7. Potential respondents contacted by email and invited via YouGov online platform (smartphone, tablet, or desktop). |
| | Advertising the survey | How/where was the survey announced or advertised? Some examples are offline media (newspapers), or | Pg. 6-7. Respondents not informed of the survey topic in the invitation email to avoid participation bias. |

| | | | |
|---|---|---|---|
| | | online (mailing lists – If yes, which ones?) or banner ads (Where were these banner ads posted and what did they look like?). It is important to know the wording of the announcement as it will heavily influence who chooses to participate. Ideally the survey announcement should be published as an appendix. | |
| **Survey administration** | Web/E-mail | State the type of e-survey (eg, one posted on a Web site, or one sent out through e-mail). If it is an e-mail survey, were the responses entered manually into a database, or was there an automatic method for capturing responses? | Pg. 6-7. Online survey via YouGov platform. Responses anonymised before the research team received them. |
| | Context | Describe the Web site (for mailing list/newsgroup) in which the survey was posted. What is the Web site about, who is visiting it, what are visitors normally looking for? Discuss to what degree the content of the Web site could pre-select the sample or influence the results. For example, a survey about vaccination on a anti-immunization Web site will have different results from a Web survey conducted on a government Web site | Pg. 6. YouGov online research panel, a nationally recognised polling platform. Quota sampling used to ensure representativeness. |
| | Mandatory/voluntary | Was it a mandatory survey to be filled in by every visitor who wanted to enter the Web site, or was it a voluntary survey? | Pg. 6-7. Voluntary survey; panel members accepted or declined the invitation. |
| | Incentives | Were any incentives offered (eg, monetary, prizes, or non-monetary incentives such as an offer to provide the survey results)? | Pg. 6-7. Standard YouGov panel incentives provided. |
| | Time/Date | In what timeframe were the data collected? | Pg. 7. Survey live from 22 November 2024 to 4 December 2024. |
| | Randomization of items or questionnaires | To prevent biases items can be randomized or alternated. | Pg. 7. Items presented in a fixed order for all respondents; no randomisation applied. |

| | | | |
|---|---|---|---|
| | Adaptive questioning | Use adaptive questioning (certain items, or only conditionally displayed based on responses to other items) to reduce number and complexity of the questions. | Pg. 8. No adaptive questioning was used. All 66 items were presented to all respondents regardless of CGM use status. |
| | Number of Items | What was the number of questionnaire items per page? The number of items is an important factor for the completion rate. | Pg. 7. 66 items total. Per-page item count not specified. |
| | Number of screens (pages) | Over how many pages was the questionnaire distributed? The number of items is an important factor for the completion rate. | Pg.7 Distributed across multiple screens; exact page count not recorded. |
| | Completeness check | It is technically possible to do consistency or completeness checks before the questionnaire is submitted. Was this done, and if "yes", how (usually JAVAScript)? An alternative is to check for completeness after the questionnaire has been submitted (and highlight mandatory items). If this has been done, it should be reported. All items should provide a non-response option such as "not applicable" or "rather not say", and selection of one response option should be enforced. | Pg. 7. Automated completeness and quality checks performed by YouGov prior to data delivery. |
| | Review step | State whether respondents were able to review and change their answers (eg, through a Back button or a Review step which displays a summary of the responses and asks the respondents if they are correct). | Pg. 7. No review step available, the YouGov platform does not permit respondents to navigate back and amend responses once submitted. |
| **Response rates** | Unique site visitor | If you provide view rates or participation rates, you need to define how you determined a unique visitor. There are different techniques available, based on IP addresses or cookies or both. | Pg. 6-7 Panel members who accepted the invitation were counted. YouGov panel methodology handles unique identification. |
| | View rate (Ratio of unique survey | Requires counting unique visitors to the first page of the survey, divided by the | Pg. 6-7. Not calculable as it was a closed YouGov panel. The total number of T2D |

| | | | |
|---|---|---|---|
| | visitors/unique site visitors) | number of unique site visitors (not page views!). It is not unusual to have view rates of less than 0.1 % if the survey is voluntary. | members initially approached was not provided by YouGov. |
| | Participation rate (Ratio of unique visitors who agreed to participate/unique first survey page visitors) | Count the unique number of people who filled in the first survey page (or agreed to participate, for example by checking a checkbox), divided by visitors who visit the first page of the survey (or the informed consents page, if present). This can also be called “recruitment” rate. | Pg. 6-7. Not calculable for the same reason as view rate. Of 553 T2D members who accepted the invitation, 531 completed the survey (completion rate among acceptors: 96.0%). |
| | Completion rate (Ratio of users who finished the survey/users who agreed to participate) | The number of people submitting the last questionnaire page, divided by the number of people who agreed to participate (or submitted the first survey page). This is only relevant if there is a separate “informed consent” page or if the survey goes over several pages. This is a measure for attrition. Note that “completion” can involve leaving questionnaire items blank. This is not a measure for how completely questionnaires were filled in. (If you need a measure for this, use the word “completeness rate”.) | Pg. 7. Overall competition rate 89.2%. T2D subsample = 96.0% completion among accepters. 3.3% did not complete, 0.7% excluded during quality checks. |
| **Preventing multiple entries from the same individual** | Cookies used | Indicate whether cookies were used to assign a unique user identifier to each client computer. If so, mention the page on which the cookie was set and read, and how long the cookie was valid. Were duplicate entries avoided by preventing users access to the survey twice; or were duplicate database entries having the same user ID eliminated before analysis? In the latter case, which entries were kept for analysis (eg, the first entry or the most recent)? | Pg. 6-7. Managed through the YouGov panel system, which controls unique participant access. Responses were anonymised before delivery to the research team. |

| | | | |
|---|---|---|---|
| | IP Check | Indicate whether the IP address of the client computer was used to identify potential duplicate entries from the same user. If so, mention the period of time for which no two entries from the same IP address were allowed (eg, 24 hours). Were duplicate entries avoided by preventing users with the same IP address access to the survey twice; or were duplicate database entries having the same IP address within a given period of time eliminated before analysis? If the latter, which entries were kept for analysis (eg, the first entry or the most recent)? | Pg. 6-7. Managed through YouGov's closed panel system, panel members have unique accounts, preventing duplicate submissions. |
| | Log file analysis | Indicate whether other techniques to analyze the log file for identification of multiple entries were used. If so, please describe. | Pg. 6-7. Log file analysis not applicable, duplicate entry prevention and response quality monitoring were managed internally by YouGov's closed panel platform. |
| | Registration | In "closed" (non-open) surveys, users need to login first and it is easier to prevent duplicate entries from the same user. Describe how this was done. For example, was the survey never displayed a second time once the user had filled it in, or was the username stored together with the survey results and later eliminated? If the latter, which entries were kept for analysis (eg, the first entry or the most recent)? | Pg. 6-7. Closed YouGov panel with registered members; each member can only participate once per survey design. |
| **Analysis** | Handling of incomplete questionnaires | Were only completed questionnaires analyzed? Were questionnaires which terminated early (where, for example, users did not go through all questionnaire pages) also analyzed? | Pg. 7. 3.3% who did not complete were excluded. 0.7% excluded during quality checks yielding a final analytical sample of 531. |
| | Questionnaires submitted with an atypical timestamp | Some investigators may measure the time people needed to fill in a questionnaire and exclude questionnaires that were | Pg. 6-7. YouGov applied standard quality checks including timing-based exclusions prior to data delivery. |

| | | | |
|---|---|---|---|
| | | submitted too soon. Specify the timeframe that was used as a cut-off point, and describe how this point was determined. | |
| | Statistical correction | Indicate whether any methods such as weighting of items or propensity scores have been used to adjust for the non-representative sample; if so, please describe the methods. | Pg. 8-9. No post-stratification weighting applied as representativeness was addressed through the quota sampling design. |

## COREQ (COnsolidated criteria for REporting Qualitative research) Checklist

A checklist of items that should be included in reports of qualitative research. You must report the page number in your manuscript where you consider each of the items listed in this checklist. If you have not included this information, either revise your manuscript accordingly before submitting or note N/A.

| Topic | Item No. | Guide Questions/Description | Reported on Page No. |
|---|---|---|---|
| **Domain 1: Research team and reflexivity** | | | |
| *Personal characteristics* | | | |
| Interviewer/facilitator | 1 | Which author/s conducted the interview or focus group? | N/A p.9 |
| Credentials | 2 | What were the researcher's credentials? E.g. PhD, MD | PhD candidate p.9 |
| Occupation | 3 | What was their occupation at the time of the study? | Full time doctoral researcher p.9-10 |
| Gender | 4 | Was the researcher male or female? | Female p.9 |
| Experience and training | 5 | What experience or training did the researcher have? | The first author had formal training in research design and qualitative methods (including coding and content/thematic analysis) analysis was conducted under supervision of experienced qualitative researchers.p.9-10 |
| *Relationship with participants* | | | |
| Relationship established | 6 | Was a relationship established prior to study commencement? | No personal relationship was established with participants prior to the study, as data were collected through an anonymous survey. P.9 |
| Participant knowledge of the interviewer | 7 | What did the participants know about the researcher? e.g. personal goals, reasons for doing the research | The survey was anonymous, and participants were not given any personal information about the researcher beyond the study information.p.9 |
| Interviewer characteristics | 8 | What characteristics were reported about the inter viewer/facilitator? e.g. Bias, assumptions, reasons and interests in the research topic | As the data were collected via survey, there was no direct interviewer. The first author's role was limited to developing the survey, including the open-ended |

| | | | |
|---|---|---|---|
| | | | questions, and later analysing responses.p.10 |
| **Domain 2: Study design** | | | |
| *Theoretical framework* | | | |
| Methodological orientation and Theory | 9 | What methodological orientation was stated to underpin the study? e.g. grounded theory, discourse analysis, ethnography, phenomenology, content analysis | A qualitative descriptive approach was used, with thematic analysis.p.11-12 |
| *Participant selection* | | | |
| Sampling | 10 | How were participants selected? e.g. purposive, convenience, consecutive, snowball | A quota sampling approach was used to ensure a nationally representative UK sample by age, gender, ethnicity, social grade, and region. This was complemented by targeted sampling of individuals with type 2 diabetes to achieve representativeness in that subgroup.p.11-12 |
| Method of approach | 11 | How were participants approached? e.g. face-to-face, telephone, mail, email | Respondents were recruited via YouGov, an international online research and analytics platform. Invitations were sent by email to panel members whose profiles matched the sampling criteria.p.9 |
| Sample size | 12 | How many participants were in the study? | 531 participants completed the survey; 259 provided a response to benefits and 181 to challenges. The remaining (91) participants did not provide analysable free-text responses to these open-ended items and were therefore not included in the qualitative analysis for those questions p.9 |
| Non-participation | 13 | How many people refused to participate or dropped out? Reasons? | Not applicable in the traditional sense. Some invited panel members did not respond to the survey invitation; among those who completed the survey, some skipped the open-ended questions. Reasons for non-response to open-ended questions were not collected. |
| *Setting* | | | |
| Setting of data collection | 14 | Where was the data collected? e.g. home, clinic, workplace | Data were collected online via a self-administered survey that participants completed in their own time and setting.p.10 |
| Presence of nonparticipants | 15 | Was anyone else present besides the participants and researchers? | N\A p.10 |

| | | | |
|---|---|---|---|
| Description of sample | 16 | What are the important characteristics of the sample? e.g. demographic data, date | The sample consisted of adults with type 2 diabetes recruited to be nationally representative by sociodemographic characteristics (age, gender, ethnicity, social grade, and region). Both CGM users and non-users were included. P.12 |
| *Data collection* | | | |
| Interview guide | 17 | Were questions, prompts, guides provided by the authors? Was it pilot tested? | Two open-ended questions were embedded within the survey. These were developed by the research team and piloted as part of the survey instrument. P.11 |
| Repeat interviews | 18 | Were repeat inter views carried out? If yes, how many? | N\A |
| Audio/visual recording | 19 | Did the research use audio or visual recording to collect the data? | N\A |
| Field notes | 20 | Were field notes made during and/or after the inter view or focus group? | N\A |
| Duration | 21 | What was the duration of the inter views or focus group? | p.9 |
| Data saturation | 22 | Was data saturation discussed? | p.9 |
| Transcripts returned | 23 | Were transcripts returned to participants for comment and/or | p.9 |
| **Topic** | **Item No.** | **Guide Questions/Description** | **Reported on Page No.** |
| | | | |
| **Domain 3: analysis and findings** | | | |
| *Data analysis* | | | |
| Number of data coders | 24 | How many data coders coded the data? | Three p01 |
| Description of the coding tree | 25 | Did authors provide a description of the coding tree? | An initial inductive code list was developed from a subset of responses and iteratively refined into a codebook. Codes were clustered into higher-order categories and organised under two overarching domains: Benefits and Challenges. Subthemes within each domain captured specific positive impacts/facilitators and barriers/concerns. No other theoretical frameworks were used to structure the coding. p.11 |
| Derivation of themes | 26 | Were themes identified in advance or derived from the data? | p.10-11 |

| | | | |
|---|---|---|---|
| Software | 27 | What software, if applicable, was used to manage the data? | Microsoft Excel was used to manage and analyse the data. p.10 |
| Participant checking | 28 | Did participants provide feedback on the findings? | No. Participants did not provide feedback on the findings. Given the anonymous online survey and panel recruitment, member-checking was not feasible. Results will be disseminated in aggregate (thesis, peer-reviewed publications/conference presentations, and a plain-language summary made publicly available). p.11 |
| *Reporting* | | | |
| Quotations presented | 29 | Were participant quotations presented to illustrate the themes/findings?<br>Was each quotation identified? e.g. participant number | Yes p.9 |
| Data and findings consistent | 30 | Was there consistency between the data presented and the findings? | Yes. Findings are grounded in the verbatim survey responses. Each theme<br>under Benefits and Challenges is illustrated with labelled quotations. p.9 |
| Clarity of major themes | 31 | Were major themes clearly presented in the findings? | Yes p.9 |
| Clarity of minor themes | 32 | Is there a description of diverse cases or discussion of minor themes? | We did not conduct quantitative content analysis . Any low-prevalence or divergent points were noted qualitatively within the relevant major theme Benefits **or** Challenges rather than presented as separate minor themes.p.9 |

Developed from: Tong A, Sainsbury P, Craig J. Consolidated criteria for reporting qualitative research (COREQ): a 32-item checklist for interviews and focus groups. *International Journal for Quality in Health Care*. 2007. Volume 19, Number 6: pp. 349 – 357

**Once you have completed this checklist, please save a copy and upload it as part of your submission. DO NOT include this checklist as part of the main manuscript document. It must be uploaded as a separate file.**
