# Supplementary material for: Perspectives on Continuous Glucose Monitoring Among Adults with Type 2 Diabetes in the United Kingdom: Cross-Sectional Survey
Source: JMIR Form Res. 2026 Jun 26;10:e89898. doi: 10.2196/89898 (PMC13354950; doi:10.2196/89898)
Supplement: Multimedia Appendix 2 [file formative_v10i1e89898_app2.docx]

­­

**Survey**

# **PART I. Characterisation**

1. **Do you live in an urban, suburban or rural area?**
2. Urban
3. Suburban
4. Rural
5. **Which UK region do you live in?**
   1. North East
   2. North West
   3. Yorkshire & the Humber
   4. East Midlands
   5. West Midlands
   6. East of England
   7. London
   8. South East
   9. South West
   10. Wales
   11. Scotland
   12. Northern Ireland
   13. Other (please specify)
6. **What is your ethnic group? (Please select one)**
7. White
8. Mixed/Multiple ethnic groups
9. Asian/Asian British/Asian Welsh
10. Black/Black British/Black Welsh/Caribbean or African
11. Another ethnic group (please specify)
12. **What is your primary language spoken at home?**
13. English
14. Welsh
15. Arabic
16. Bengali
17. French
18. Gujarati
19. Italian
20. Panjabi
21. Polish
22. Portuguese
23. Romanian
24. Spanish
25. Urdu
26. Chinese
27. Other (please specify)
28. What is your current employment status?
29. Full-time employment
30. Part-time employment
31. Volunteer
32. Not currently working
33. Student
34. Retired
35. Other (please specify)
36. **What sex were you assigned at birth?**
37. **Male**
38. **Female**
39. **Prefer not to say**
40. **What is your religious affiliation? (Please select one)-**
41. Buddhist
42. Christian
43. Hindu
44. Jewish
45. Muslim
46. Atheist
47. Sikh
48. Other (please specify)
49. Prefer not to say
50. **What is the highest level of education you have completed?**
51. Primary education
52. Secondary education (e.g., GCSEs or equivalent)
53. Further education (e.g., A levels, NVQ Level 2 or 3)
54. Higher education (e.g., undergraduate degree or higher)
55. Other qualifications (such as professional or vocational qualifications)
56. **What is your household's total annual income before tax? (Please enter the amount in GBP)**

£_____________

1. How often do you meet with family or friends?
2. Daily
3. Weekly
4. Monthly
5. Rarely
6. Never
7. Do you have any disabilities?
8. Yes
9. No

**The next questions aim to access how comfortable you feel using digital technologies**

|  | Strongly agree | Agree | Neutral | Disagree | Strongly disagree |
| --- | --- | --- | --- | --- | --- |
| 1. I **know how to find helpful health resources on the internet** |  |  |  |  |  |
| 1. **I know how to use the internet to answer my health questions** |  |  |  |  |  |
| 1. **I know what health resources are available on the internet** |  |  |  |  |  |
| 1. **I know where to find helpful health resources on the internet** |  |  |  |  |  |
| 1. **I know how to use the health information I find on the internet to help me.** |  |  |  |  |  |
| 1. **I have the skills I need to evaluate the health resources I find on the internet** |  |  |  |  |  |
| 1. **I can tell high-quality health resources from low-quality health resources on the internet** |  |  |  |  |  |
| 1. **I feel confident in using information from the internet to make health decisions** |  |  |  |  |  |

1. Do you have any chronic health conditions? (Check all that apply)
2. Mobility impairment
3. Visual impairment
4. Hearing impairment
5. Mental health condition
6. Type 1 Diabetes
7. Type 2 Diabetes
8. Cardiovascular disease (e.g. heart disease)
9. Respiratory condition
10. Chronic pain
11. Other (please specify)

***< Logic: If answering yes to T2D, please show Q 21-24 >***

1. For how long did you have Type 2 Diabetes?
2. Less than 1 year
3. 1-2 years
4. 3-5 years
5. More than 5 years
6. **Are you currently taking medication for Type 2** **Diabetes?**
7. Yes, oral antidiabetic drugs (e.g. metformin)
8. Yes, insulin
9. Yes, oral antidiabetic drugs + insulin
10. No
11. **Do you have any diabetes-related complications? (Select all that apply)**
12. Cardiovascular disease
13. Neuropathy (nerve damage)
14. Retinopathy (eye damage)
15. Nephropathy (kidney damage)
16. Foot problems (ulcers, infections)
17. Other (Please specify) [Text Box]
18. How often do you visit your healthcare provider for diabetes-related care?
19. Weekly
    1. Bi-weekly
    2. Monthly
    3. Every 3 months
    4. Every 6 months
    5. Annually
    6. Other (please specify): ____________

# **PART II. Use of DHTs for self-management**

**In this section, you will be asked about how you use Digital Health Technologies (DHTs) for self-management. These questions are designed to understand your engagement with DHTs in managing your health & wellness. Your responses will help us assess the role of DHTs in supporting self-management practices & improving health outcomes. Please answer the following questions based on your personal experiences & usage of these technologies.**

|  | Health & fitness app | Continuous Glucose Monitoring (CGM) devices (These are ‘patches’ that stick to your arm & can read the concentration of glucose in the blood). | Other wearable devices for health monitoring & tracking  (e.g. Fitbit, Apple Watch | Online symptom checkers  (e.g., WebMD Symptom Checker Healthily,  & Mayo Clinic Symptom Checker) | Health information website (e.g., NHS websites, WebMD) | Social media (e.g., Instagram, TikTok, Facebook) |
| --- | --- | --- | --- | --- | --- | --- |
| 1. I am aware of the following digital health tools for self-management [Knowledge] |  |  |  |  |  |  |
| 1. I have used the following digital health tools for self-management [Attitudes] |  |  |  |  |  |  |
| 1. I feel confident in using the following digital health tools for self-management [Practice] |  |  |  |  |  |  |
| COM-B | | | | | | |
| 1. I feel capable of using these technologies in principle |  |  |  |  |  |  |
| 1. I feel I have sufficient opportunity to use these technologies for self-management |  |  |  |  |  |  |
| 1. I feel I have enough motivation to use these technologies for self-management |  |  |  |  |  |  |
| UTAUT2 | | | | | | |
| Performance Expectancy | | | | | | |
| 1. I find these technologies useful for self-management |  |  |  |  |  |  |
| 1. These technologies improve control over my own health |  |  |  |  |  |  |
| Effort Expectancy | | | | | | |
| 1. Learning how to use these technologies is easy for me. |  |  |  |  |  |  |
| Social Influence | | | | | | |
| 1. People who are important to me think that I should use these technology |  |  |  |  |  |  |
| Facilitating Condition | | | | | | |
| 1. I have resources necessary to use this technology |  |  |  |  |  |  |
| 1. I have the knowledge necessary to use this technology |  |  |  |  |  |  |
| 1. I can get help from others when I have difficulties using this technology |  |  |  |  |  |  |
| Hedonic Motivation | | | | | | |
| 1. Using this technology is enjoyable |  |  |  |  |  |  |
| Habit | | | | | | |
| 1. The use of this technology has become a habit for me |  |  |  |  |  |  |
| Behavioural Intention (users) | | | | | | |
| 1. I intend to continue using this technology in the future |  |  |  |  |  |  |
| Behavioural intention (non-user) | | | | | | |
| 1. I intend to try these technologies |  |  |  |  |  |  |
| Perceived trust | | | | | | |
| 1. I trust this technology |  |  |  |  |  |  |
| Privacy Concerns | | | | | | |
| Perceived Surveillance | | | | | | |
| 1. I am concerned that this technology may monitor my activity |  |  |  |  |  |  |
| Perceived Intrusion | | | | | | |
| 1. I feel that as a result of my using this technology, information about me is out there that, if used, will invade my privacy. |  |  |  |  |  |  |
| Secondary use of personal information | | | | | | |
| 1. I am concerned this technology may use my personal information for other purposes without notifying me or getting my authorisation. |  |  |  |  |  |  |

**CGM BLOCK**

1. Have you heard of Continuous Glucose Monitoring (CGMs)? These are ‘patches’ that stick to your arm & can read the concentration of glucose in the blood?

- Yes
- No

***< Logic: If answering yes to 46, please show Q 47-52 >***

1. How did you learn about CGMs (Check all that apply)
2. Personal research
3. Advice from healthcare providers
4. Information from diabetes education programs
5. Experiences of family or friends
6. Online forums or communities
7. Other (Please specify)
8. Have you ever used CGMs

- Yes (*complete this block & also complete the next)*
- No *(Exit this block & do not complete the next)*

1. If yes, for how long did you use CGM?
2. Less than a month
3. 1-3 months
4. 4-6 months
5. More than 6 months
6. For which purpose, have you primarily used CGM? (Select all that apply)
7. Monitoring glucose levels
8. Weight management
9. General health monitoring
10. Sports performance optimization
11. Support with dietary choices & exercise
12. Early detection of potential glucose metabolism issues
13. Other (please specify): __________
14. Do you currently share your CGM data with your healthcare provider?

- Yes
- No

If yes

1. How do you currently share your CGM data with your healthcare provider?
   (Please select all that apply)
2. I share my data through a CGM app linked to my provider's system.
3. I email or message my CGM data to my healthcare provider.
4. I bring printed reports or screenshots to appointments.
5. My healthcare provider downloads the data during my visits.
6. I do not share my CGM data with my healthcare provider.
7. Other (please specify)

Regarding perceived impact with CGM use, to what extent do you agree with the following statements:

|  | Completely disagree | Disagree | Not agree nor disagree | Agree | Completely agree |
| --- | --- | --- | --- | --- | --- |
| 1. CGM has improved my communication with healthcare providers? |  |  |  |  |  |
| 1. CGM use makes me feel more empowered to manage my health |  |  |  |  |  |
| 1. CGM has improved my understanding of my body’s response to different foods & activities |  |  |  |  |  |
| 1. CGM use influenced has improved my health management? |  |  |  |  |  |
| 1. CGM use has improved my specific health markers (e.g., HbA1c levels) |  |  |  |  |  |
| 1. CGM has reduced my need for visits/appointments |  |  |  |  |  |
| 1. CGM has reduced my use of other NHS resources (i.e. glycaemic control tests) |  |  |  |  |  |
| 1. CGM has improved my safety by allowing me to better prevent very high / very low glucose events |  |  |  |  |  |
| 1. CGM use has allowed me to have quicker access to care when I needed it |  |  |  |  |  |
| 1. CGM use has allowed me overcome inequities in access to care |  |  |  |  |  |

1. Do you make adjustments to your diet based on CGM readings?
2. Always
3. Often
4. Sometimes
5. Rarely
6. Never
7. Do you make adjustments to your physical activity based on CGM readings?
8. Always
9. Often
10. Sometimes
11. Rarely
12. Never
13. In your perspectives, which are the main strengths and opportunities of using Continuous Glucose Monitoring (CGM)? (Free text)
14. your perspectives which are the main weaknesses and threats to using Continuous Glucose Monitoring (CGM)? (Free text)

**CONSENT TO CONTACT BLOCK**

Thank you for completing this survey! As part of this study, we are also conducting interviews to gain a deeper understanding of the experiences and perspectives regarding CGM in self-management. The interviews will take approximately 35-45 minutes and will be conducted virtually. If you are interested in participating in the interview portion of this study, please provide your contact information below. Your contact details will only be used for scheduling the interview and will not be linked to your survey responses, ensuring your anonymity in the survey data.

- Name (optional):
- Email Address:

Note: By providing your contact information, you are expressing interest in the interview study. You will be contacted by a member of the research team to schedule a convenient time for the interview. Participation in the interview is entirely voluntary, and you may choose to withdraw at any time.

If you have any questions or require further information, please feel free to reach out to the research team at [a.alharbi23@imperial.ac.uk](mailto:a.alharbi23@imperial.ac.uk)

END
